# Alterations in the Retinal Nerve Fiber Layer Thickness Color Map in Non- Glaucomatous Eyes with Myopia

**DOI:** 10.4274/tjo.galenos.2020.58726

**Published:** 2021-02-25

**Authors:** Hayati Yılmaz, Mehmet Talay Köylü, Yağmur Seda Yeşiltaş, Dorukcan Akıncıoğlu, Duygu Yalınbaş, Yeşim Gedik Oğuz, Atilla Bayer, Fatih Mehmet Mutlu

**Affiliations:** 1 University of Health Sciences Turkey, Ümraniye Training and Research Hospital, Clinic of Ophthalmology, İstanbul, Turkey; 2 University of Health Sciences Turkey, Gülhane Faculty of Medicine, Department of Ophthalmology Ankara, Turkey; 3 Atatürk State Hospital, Antalya, Turkey; 4 Dünyagöz Hospital, Ankara, Turkey

**Keywords:** Myopia, retinal nerve fiber layer, glaucoma

## Abstract

**Objectives:**

To determine the normal values for retinal nerve fiber layer thickness (RNFLT) in myopic patients without glaucoma and analyze the changes in their color map.

**Materials and Methods:**

A total of 245 eyes without glaucoma were included in the study. According to the degree of myopia, the cases were divided into 4 groups: control group (+1.00/-1.00 D; n=70), Group 1 (-1.00/-3.00 D; n=50), Group 2 (-3.00/-6.00 D; n=75), and Group 3 (>-6.00 D; n=50). Intra-group comparisons were performed in terms of superotemporal, superonasal, nasal, inferonasal, inferotemporal, temporal, and global RNFLT (Heidelberg Spectralis, Optic Coherence Tomography, Germany) and the color coding of these quadrants (green: within normal limits, yellow: borderline, red: outside normal limits).

**Results:**

All groups were similar in age and gender (p>0.05). As the degree of myopia increased, RNFLT became thinner in the upper and lower temporal and upper and lower nasal quadrants (p<0.01). The rate of measurements considered borderline and outside normal limit in at least 1 quadrant was higher in groups with higher myopia for all quadrants (p<0.05). This rate was found to be 8/70 (11.4%) for the control group, 9/50 (18.0%) for Group 1, 21/75 (28.0%) for Group 2, and 33/50 (66.0%) for Group 3 (p<0.01).

**Conclusion:**

The high rate of RNFLT classified as borderline or outside normal limits in myopic patients is a finding to which clinicians should pay attention in order not to make a misdiagnosis, especially in cases of suspected glaucoma.

## Introduction

Myopia, known to affect approximately 1.6 billion people worldwide, is one of the most common refractive errors and is considered to be an epidemic with increasing prevalence.^1^ The risk of glaucoma is known to increase in myopic eyes and in advanced age.^2,3^ However, glaucomatous changes in myopic eyes are difficult to detect.^4^ Especially structural changes, such as wide or tilted optical discs and myopia-related gamma and delta zones caused by myopia, result in ophthalmoscopic examination being insufficient in detecting anatomical glaucomatous changes. Furthermore, structural and functional tests used for glaucoma diagnosis can be obscured by myopia.^4^

Analysis of retinal nerve fiber layer thickness (RNFLT) plays an important role in glaucoma management.^5,6,7^ However, because the kappa angle between the temporal vascular structures decreases in inverse proportion to the axial length (AL) in myopic eyes, the superior and inferior temporal retinal nerve fiber layers (RNFL) are located closer to each other as they approach the temporal quadrant.^8^ In the light of this information, it is clear that color coding used by software in the analysis of normative data (green: within normal limits, yellow: borderline, red: outside normal limits) can also lead to the misinterpretation of RNFLT data. In this study, we aimed to evaluate how currently known changes in RNFLT measurements in myopic eyes affect this color coding.

## Materials and Methods

This study was carried out in accordance with the principles of the Helsinki Declaration after obtaining the approval of the Non-Interventional Clinical Research Ethics Committee of the Health Sciences University, Turkey. The files of the patients that presented to the cornea and refractive surgery and glaucoma outpatient clinics of Gülhane Training and Research Hospital Ophthalmology Department between June 2019 and January 2020 were retrospectively screened. Patients older than 18 years with emmetropia and axial myopia without glaucoma (intraocular pressure [IOP] <21 mmHg, cup/disc ratio <0.4), who had undergone a complete ophthalmologic examination including autorefraction (Tonoref III, Nidec Co. Ltd, Aichi, Japan), best corrected visual acuity (BCVA) with Snellen chart, IOP measurement by an air puff tonometer (Tonoref III, Nidec Co. Ltd, Aichi, Japan), slit lamp biomicroscopy, dilated fundus examination, AL measurement (AL-SCAN, Nidek Co. Ltd, Aichi, Japan), and optic coherence tomography (OCT; Spectralis OCT Heidelberg Engineering, Heidelberg, Germany) for RNFLT analysis were included in the study. Patients with suspected glaucoma had also undergone visual field (VF) testing (Humprey Field Analyzer II, Carl Zeiss Meditec, Inc.). Excluded from the study were patients with hyperopic eyes having a spherical equivalent (SE) greater than +1.00 D and myopic eyes other than axial myopia, those with ocular pathologies (cataract, glaucoma, pigmentary dispersion syndrome, pseudoexfoliation syndrome, retinal diseases, and corneal diseases such as dry eye and >3D astigmatism) or ocular surgery history, and those with systemic diseases that could cause changes in ocular physiology (diabetes mellitus, connective tissue diseases, and autoimmune diseases). The international myopic maculopathy classification and grading system was applied to all myopic patients to evaluate fundus changes.^9^ According to this grading system, patients with category 0 (no myopic degeneration) and 1 (mosaic “tessellated” fundus) were included in the study while those in the other myopic eye categories were excluded. According to their degree of myopia, the cases included in the study were divided into 4 groups: the control group (+1.00/-1.00 D; n=70), Group 1 (-1.00/-3.00 D; n=50), Group 2 (-3.00/-6.00 D; n=75), and Group 3 (>-6.00 D; n=50). For the control group, the right eye was evaluated. For the remaining groups, the right eye was evaluated in bilateral myopia cases and the myopic eye in unilateral cases.

### RNFLT Analysis

**Figure 1:**
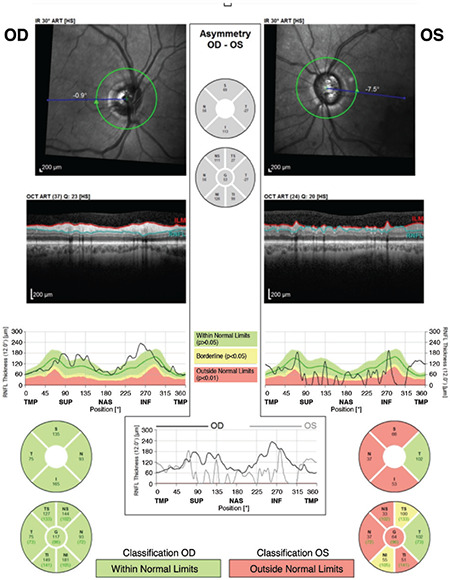
Retinal nerve fiber layer (RNFL) analysis output of a patient OD: Right eye, OS: Left eye

Spectralis OCT (version 4.0) (Heidelberg Engineering, Heidelberg, Germany) was used for the measurement of RNFLT. This device has an A-scan rate of 40,000/s using a light source of 820 nm. For each eye with non-dilated pupilla, an en face image focusing on the optic nerve head (ONH) was generated using a confocal scanning laser ophthalmoscope, and after a 3.4 mm circle was centered on ONH, 15 images were acquired under high-resolution settings and averaged automatically by built-in software. The scanning was repeated if the signal strength was lower than 15 dB. For each case, the global RNFLT and the RNFLT values of the superotemporal, superonasal, nasal, inferonasal, inferotemporal, and temporal quadrants were recorded. In addition, the color codes automatically generated by the device software (green: within normal limits [less than 95% of individuals in the age and basal membrane opening area-adjusted reference database have RNFLT greater than the measured value], yellow: outside the 95% normal limits [borderline], and red: outside the 99% normal limits) were recorded (Figure 1).

### Statistical Analysis

SPSS v. 21 (IBM Corp., Armonk, NY, USA) was used for statistical analysis. Quantitative variables were defined as mean and standard deviation (SD) and qualitative variables as percentages. The Shapiro-Wilk test was used to evaluate whether the sample came from a normally distributed population. According to the results of the normality analysis, RNFLT values were compared between the groups using parametric ANOVA or non-parametric Kruskal-Wallis tests and post-hoc analysis (Tukey for ANOVA and pairwise comparisons for Kruskal-Wallis). The differences between the groups in terms of the color codes of the quadrants were determined by the chi-square test and post-hoc Bonferroni analysis. Generalized linear models (GLM) were used to search for associations between RNFLT and AL. A different GLM was created with each RNFLT value as a dependent factor. Sex was selected as a factor, age and AL were selected as covariates, and the main effects of AL on RNFLT were calculated. After these calculations, we added study groups as a factor to these same models and the associations between RNFLT and AL independent of the study groups were determined again. GLM results were given with correlation coefficient (B), lower and upper bounds of 95% Wald confidence interval (CI) and p-value. A p-value less than 0.05 was considered statistically significant.

## Results

The mean age of the patients included in the study was 26.04±5.51 years in the control group, 26.16±7.80 years in Group 1, 26.30±6.80 years in Group 2, and 27.84±4.34 years in Group 3 (p=0.542). There was no significant difference between the groups in terms of sex (p=0.324), IOP (p=0.436), or BCVA (p=0.232). The mean AL was 22.96±0.23 mm in the control group and 23.54±0.53, 24.12±0.51, and 24.94±0.42 mm in Groups 1 to 3, respectively. The mean AL significantly differed according to the paired comparisons of the groups (p<0.001). The mean difference in the SE values was -0.32±0.25 D for the control group and -1.62±0.46, -4.34±1.01, and -7.65±1.45 D in Groups 1 to 3, respectively. The groups also significantly differed in relation to SE values (p<0.001). The demographic and clinical features of the study subjects are presented in Table 1.

### Results of RNFLT Analysis

The mean and quadrant RNFLT values of the groups are given in Table 2. While there was no significant difference in the RNFLT of the nasal and temporal quadrants between the groups, the RNFLT of all the remaining quadrants and the mean values significantly differed (p<0.05). According to the paired comparisons, the RNFL in the superotemporal quadrant was significantly thinner in Group 3 compared to the other groups (p<0.001 for control group Group 3, p=0.004 for Group 1, 3, and p=0.004 for Groups 2, 3). Concerning the RNFLT of the superonasal quadrant, no significant difference was observed between the control group and 1 (p=0.076) or between Group 2 and 3 (p=0.126) but the control group and Group 1 were found to have significantly thicker superonasal RNFL than Groups 2 and 3 (p<0.001). In the inferonasal quadrant, the control group had a similar RNFLT to Group 1 but significantly thicker RNFLT than Groups 2 and 3 (p=0.257, 0.003 and 0.001, respectively). The remaining paired comparisons revealed no significant difference in the RNFLT of the inferonasal quadrant (p>0.05). In the inferotemporal quadrant, the control group had a significantly thicker RNFLT compared to Groups 1 to 3 (p=0.006, <0.001 and <0.001, respectively). When the global RNFLTs were compared, the RNFLT of the control group was similar to Group 1 (p=0.092), and significantly thicker than Groups 2 and 3 (p=0.002 and <0.001, respectively).

The associations between RNFLT and AL are presented in Table 3. RNFLT measurements were strongly associated with AL in all quadrants except nasal and temporal. The associations disappeared after including the groups (the degree of myopia) into the GLMs.

The color codes corresponding to the RNFLT values of all quadrants are given in Table 4. In the inter-group comparisons using the chi-square test and Bonferroni method, the color distribution among the groups was similar for the temporal quadrant (p=0.316) but a significant difference was observed between the groups in all other quadrants (p<0.05). In Group 3, the number of areas with a green code in the superotemporal and superonasal quadrants was significantly lower while the number of those with a red code in these quadrants was significantly higher compared to the other groups (p<0.05). There was no significant difference between the groups in terms of the number of yellow codes (p>0.05). The number of areas receiving a green code in the nasal quadrant was significantly higher in the control group and Group 1 compared to Groups 2 and 3, whereas the opposite was seen in relation to the yellow code (p<0.05). There was no difference between the groups in terms of the number of red-coded areas in the nasal quadrant (p>0.05). The number of those coded green in the inferonasal quadrant was significantly lower in Group 3 than in the other groups while the opposite was true for the number of red- and yellow-coded areas (p<0.05). The number of green-coded areas in the inferotemporal quadrant was significantly higher in the control group and Group 1 than in Groups 2 and 3 (p<0.05). In addition, in the inferotemporal quadrant, the number of those coded green was higher in Group 2 than in Group 3, and the opposite was observed in terms of the number of areas with yellow or red codes (p<0.05). In the comparison of the groups, the number of areas with a green code was significantly lower in Group 3 compared to the other groups, and the opposite was true for the number of those coded red (p<0.05). In terms of the mean RNFLT color codes, the number of yellow-coded cases was significantly less in the control group and Group 1 compared to Groups 2 and 3 (p<0.05).

## Discussion

The analysis of RNFLs plays a very important role in glaucoma management, and myopia can affect this assessment and lead to false evaluations.^6,8,10^ Therefore, the variation of RNFLT from normative data in myopic eyes and the change in RNFLT caused by myopia have been investigated by many researchers.^11,12,13,14^ In the current study, unlike previous research, in addition to the interaction between myopia and RNFLT, we investigated the differences in the color codes of RNFLT in myopic eyes compared to emmetropes using color maps, which are frequently utilized by ophthalmologists in routine clinical practice.

RNFLT values were found to be strongly associated with AL. However, the associations disappeared after performing statistical analyses independent from the study groups, which were determined in accordance with SE, indicating highly significant collinearity between SE and AL. This result was expected because only axial myopia patients were included in the present study. Therefore, we only used SE and the study groups for further analyses.

In this study, as SE increased in myopic patients, the number of eyes receiving yellow and red codes increased, especially in the superotemporal, superonasal, inferotemporal, and inferonasal quadrants. Among the patients with a lower SE, there was a higher number of green-coded cases in the nasal quadrant and a lower number of yellow-coded cases while there was no significant difference in relation to the red code. In the temporal quadrant, the difference between the groups was not statistically significant. These findings were consistent with the RNFLT values. In their research in healthy myopic eyes, Leung et al.^8^ and Yamashita et al.^10^ suggested that as a result of the more temporal location of the upper and lower peaks of the RNFLT profile, RNFLT is perceived to be abnormally thick in the temporal quadrant and abnormally thin in the inferior and superior quadrants compared to the normative data, leading to false positive evaluations, while RNFL defects may be overlooked in the temporal quadrant. Furthermore, Özdek et al.^15^ showed that there was thinning in RNFL in the superior and inferior quadrants in correlation with increasing myopia values. In our study, different from previous researchers, we detected thicker RNFL values with a higher SD in cases with higher SE not only in the temporal quadrant but also the nasal quadrant.

“Red disease” refers to a pseudo-condition in which RNFLT measured by OCT is indicated as outside normal limits. It is often caused by high myopia, mainly due to pseudo-thinning of the RNFL which is stretched to cover the elongated globe.^16,17^ Schuman^16^ and Chong and Lee.^17^ have suggested using macular OCT data for glaucoma screening in patients with high axial myopia and stated that the causes of this OCT abnormality are negligible up to -10 D. In our study, there was no subject over -10 D. However, red disease should always be kept in mind while evaluating myopic patients for glaucoma. RNFL and macular OCT data should be analyzed simultaneously even in patients with lower degrees of myopia because, as in our study, myopic patients had more sectors in which RNFLT profiles are outside normal limits.

It is known that myopia is a risk factor for glaucoma.^18,19,20^ Myopic eyes have a higher cup-disk ratio, thinner RNFL, and higher mean deviation in the VF analysis compared to eyes without refractive error.^15,21^ VF and RNFLT analyses are very important in distinguishing whether the defects detected are due to glaucoma or myopia. In addition, it is known that defects caused by a tilted optic disc or stable high myopia do not progress over time.^22^ The absence of progression in followed-up cases may be a sign that defects are caused by myopia or optical disc structure. However, it should be remembered that degenerative myopia may progress in VF and RNFL defects over time. In such cases, it is essential to record the changes in the fundus.

It can be predicted that myopia, an epidemic affecting an ever-increasing number of people worldwide, will further complicate the future diagnosis of glaucoma, which is one of the leading causes of vision loss and blindness. In primary open-angle glaucoma cases, Dursun et al.^23^ showed a significant correlation between VF indices and OCT findings and suggested that the follow-up of RNFLT provided earlier indications of progression for early diagnosis than VF analysis. RNFL analysis is gradually becoming the gold standard in the follow-up of glaucoma, especially in the early stage.^24^

### Study Limitations

In this study, we only included patients classified as category 0 and category 1 according to the international myopic maculopathy classification and grading system. Different results may have been obtained if patients with higher degrees of myopia had been evaluated. Therefore, in future studies, it will be more appropriate to evaluate different subgroups according to the degree of myopic degeneration. Another limitation of the study can be considered its cross-sectional and retrospective design. VF testing was not executed for all of the subjects, only the patients with suspected glaucoma underwent VF testing; therefore, there is a possibility that some patients could have normal-tension glaucoma or pre-perimetric glaucoma. Long-term prospective follow-up studies are needed to exclude all the patients with glaucoma and determine whether there is progression of changes in the RNFLT values of myopic eyes over time.

## Conclusion

In conclusion, in RNFL analysis, which plays an essential role in glaucoma diagnosis and follow-up, it is important to know that there are thinning and yellow- and red-coded areas in the color map caused by myopia while no change or thickening is observed in the temporal quadrant. In addition, it should be kept in mind that SD may be high in myopic patients due to the differences in the structure of the optic disc; therefore, there is a need to include the SE in the calculation in cases suspected of glaucoma.

## Figures and Tables

**Table 1:**
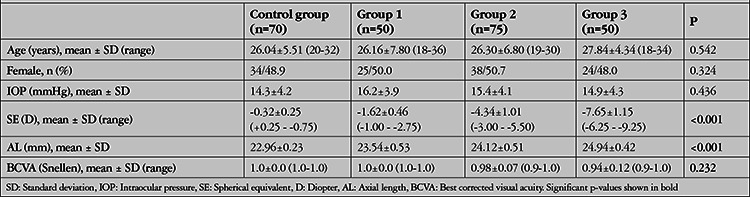
Demographic and clinical features of the study subjects

**Table 2:**
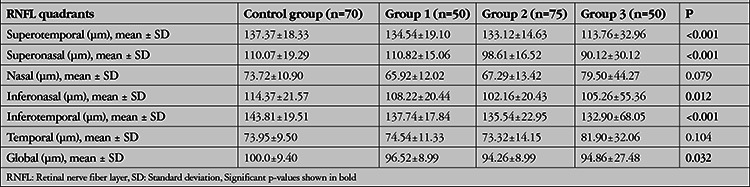
Retinal nerve fiber layer (RNFL) thickness results of the subjects

**Table 3:**
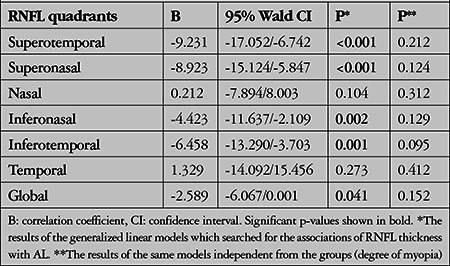
Associations between quadrant retinal nerve fiber layer (RNFL) thicknesses and axial length (AL) measurement

**Table 4:**
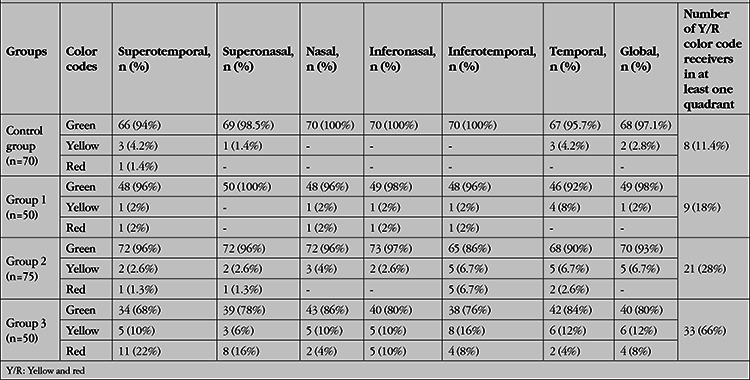
The color map results of the corresponding retinal nerve fiber layer (RNFL) quadrants
